# Comparison of the Treatment Efficacy of Continuous Renal Replacement Therapy and Intermittent Hemodialysis in Patients With Acute Kidney İnjury Admitted to the Intensive Care Unit

**DOI:** 10.7759/cureus.21707

**Published:** 2022-01-29

**Authors:** Fatma Yılmaz Aydın, Emre Aydın, Ali Kemal Kadiroglu

**Affiliations:** 1 Internal Medicine, Dicle University, Diyarbakır, TUR; 2 Nephrology, Dicle University, Diyarbakır, TUR; 3 Nephrology: Intensive Care, Dicle University, Diyarbakır, TUR

**Keywords:** mortality, intensive care unit, intermittent hemodialysis, continuous renal replacement therapy (crrt), renal replacement therapy (rrt), acute kidney injury

## Abstract

Introduction and aim

Acute kidney injury (AKI) is part of the multiple organ dysfunction syndrome in critically ill patients and is a common condition in intensive care units (ICUs). Renal replacement therapy (RRT) is the cornerstone of treatment for AKI in critically ill patients. This patient population has a high mortality rate despite RRT. There are two methods of RRT for patients in ICUs: intermittent hemodialysis (IHD) and continuous renal replacement therapy (CRRT). Both CRRT and IHD similarly provide adequate metabolic control. We aimed to compare these two RRT modalities in terms of ICU stay, mortality, and laboratory recovery in these patients with high mortality.

Materials and methods

A total of 120 patients with AKI who needed RRT in the ICU were included in the study (CRRT, n:40; IHD, n:80). Acute Physiology and Chronic Health Evaluation (APACHE) II, Sepsis-related Organ Failure Assessment (SOFA), and Simplified Acute Physiology Score (SAPS)-II scores at the time of admission to the ICU were calculated. Mean arterial pressure, urea, creatinine, sodium, potassium, calcium, pH, lactate, and bicarbonate levels were measured before and after dialysis. Patients were classified as living and deceased. Factors affecting the length of stay in the intensive care unit and 30-day mortality were evaluated. The variability in laboratory parameters between groups before and after dialysis was examined. The groups were compared with these parameters.

Results

Sixty-one point seven percent (61.7%, n:74) of the patients were female. The mean age was 62.90±13.64 years. At the time of admission to the ICU, the patients' SAPS II score was 45.05±12.76, APACHE II score was 22.05±6.32, and SOFA score was 8.26±2.48. 66.7% (n:80) of the patients included in the study died, and the length of stay of these patients in the ICU was 12.85±10.23 days. When the groups were compared, SAPS II, APACHE II scores, and SOFA scores were significantly higher in the CRRT group than in the IHD group (p:0.038, p:0.015, p:0.027, respectively). Although the length of stay in the ICU was shorter in the CRRT group, it was not statistically significant (p:0.075). There was no statistically significant difference between the groups in terms of mortality (p: 0.891). SAPS-II, APACHE II, and SOFA score affected 30-day mortality while age, gender, and RRT modalities were not associated with mortality. The improvement in laboratory parameters between the pre and post-RRT groups was statistically more significant in the IHD group (p<0.001). It was determined that there was a statistically greater decrease in mean arterial pressure in the IHD group (p<0.001).

Conclusions

It was determined that there was no difference between the CRRT and IHD modalities applied in patients with AKI admitted to the ICU in terms of mortality and length of stay in the ICU. It was observed that both modalities improved on laboratory parameters, but the improvement was greater in the IHD group. However, it was determined that there was a statistically greater decrease in mean arterial pressure in the IHD group.

## Introduction

Acute kidney injury (AKI) is one of the important complications that occur in critically ill patients followed in the intensive care unit (ICU), and it is an independent risk factor for mortality [1]. Although it varies according to the population studied, AKI develops in 30-60% of patients followed in the ICU, approximately 20% of patients with AKI, and approximately 5% of all ICU patients require renal replacement therapy (RRT) [2-3]. The mortality rate in AKI patients requiring RRT varies between 40% and 55% [1].

RRT is the main complementary therapy of severe AKI in critically ill patients. On the basis of all RRT modalities, it is aimed to remove the liquid-solute load and to provide acid-base balance [4]. There are two methods of RRT for patients in the ICU: intermittent hemodialysis (IHD) and continuous renal replacement therapy (CRRT). With IHD applied for an average of three to four hours, the removal of fluid and solute loads and the approximation of the acid-base disturbance to normal is higher. However, excessive fluid withdrawal in critically ill patients may exacerbate hemodynamic instability by causing more hypotensive episodes [4]. CRRT, which can be applied from 24 hours to several days, provides a slow but continuous removal of fluid and solute load, providing better hemodynamic stability [5].

The choice of RRT modality is usually determined by the patient's hemodynamic condition. CRRT is used when patients are hemodynamically unstable [6]. In addition, CRRT is recommended in critical patients with generalized brain edema, acute brain injury, increased intracranial pressure, and AKI and/or multiorgan failure [5]. In critically ill patients who develop AKI with hyperkalemia, rhabdomyolysis, and intoxication, IHD is preferred because the solute loads need to be removed quickly [4].

There are studies comparing CRRT and IHD in the literature [5-7]. It is controversial which of these two modalities gives better results on patient survival and clinical and laboratory parameters. Various groups have compared these two methods, but these studies are often non-randomized and retrospective. In this prospective study, we investigated the treatment efficacy of IHD and CRRT for the treatment of AKI in critically ill patients admitted to the ICU. Our study objectives were to compare the effects of RRT modality on ICU length of stay, mortality, and clinical and laboratory outcomes after RRT.

## Materials and methods

A total of 120 AKI patients, over the age of 18 who needed RRT in the tertiary ICU between April 2017 and April 2020, were included in our study, regardless of gender. AKI was diagnosed according to Kidney Disease: Improving Global Outcomes (KDIGO) criteria [8]. Among the RRT modalities, CRRT (n:40) was applied to patients with hemodynamic instability, and IHD (n:80) was applied to hemodynamically stable patients. As an indication of RRT, uremic symptoms (nausea-vomiting, neurological complications, pericardial effusion), hyperkalemia unresponsive to medical therapy, volume overload unresponsive to diuretic therapy, severe metabolic acidosis (pH<7.2), and urinary output of less than 0.5 ml/kg for 12 hours despite the correction of the volume deficit was defined as the presence of at least one of the criteria. Patients with previous chronic kidney disease and end-stage renal disease were excluded from the study. The Acute Physiology and Chronic Health Evaluation (APACHE) II score, Simplified Acute Physiology Score (SAPS)-II, and Sepsis-related Organ Failure Assessment (SOFA) score were calculated during hospitalization. Mean arterial pressure (MAP), urea, creatinine, sodium, potassium, calcium, pH, lactate, and bicarbonate levels were measured before and after dialysis. Patients were classified as living and deceased. Length of stay in the intensive care unit and 30-day mortality were calculated. The groups were compared with these parameters. For IHD, initial hemodialysis was scheduled for two hours. It was planned as four hours in later indications (AK98, Gambro, Lund, Sweden). For CRRT, the duration of treatment was planned to be not less than 24 hours according to the indication. Continuous venovenous hemodiafiltration (CVVHDF) at a dose of 30 ml/kg/hour was preferred. Device settings were adjusted according to the patient's hemodynamic, clinical, and laboratory values (Prismaflex, Baxter, Glenview, Illinois). Laboratory tests were taken from all patients before and one hour after RRT. The study was approved by the local ethics committee of Dicle University Faculty of Medicine (12.01.2017 / 12) and was funded by Dicle University Scientific Research Project (DUBAP) numbered 17TF007.

Statistical analysis

Statistical analyzes of the results obtained in the study were performed using the Statistical Package for Social Sciences (SPSS) 24 program (IBM Corp., Armonk, NY). Descriptive statistics were used for demographic data. The conformity of the variables to the normal distribution was examined by visual (histogram and probability graphs) and analytical methods (Kolmogorov- Smirnov/Shapira-Wilk tests). Results were given as numbers and percentages for categorical variables and as mean ± standard deviations for continuous variables. An independent sample t-test was used as a parametric test for those with normal distribution, and the Mann-Whitney U test was used as a non-parametric test for those who did not show normal distribution. The comparison of the data of the groups was made using the chi-square and Fisher test. The Kaplan-Meier method was used for survival analysis and compared using log-rank analysis. The Cox Regression analysis was used for the variable analysis of mortality. To evaluate the changes in the parameters before and after dialysis, the difference of the values ​​was taken, the independent sample t-test was applied as a parametric test for those with normal distribution, and the Mann-Whitney U test was applied as a non-parametric test for those who did not show normal distribution. A p-value of less than 0.05 was considered statistically significant.

## Results

One hundred twenty patients who were followed up in the ICU and underwent RRT for AKI were included in our study. Of this patient population, 80 patients underwent IHD and 40 patients underwent CRRT. Sixty-one point seven percent (61.7%; n:74) of the patients were female and 38.3% (n:46) were male. The mean age was 62.90±13.64 years. At the time of admission to the ICU, the patients' SAPS-II was 45.05±12.76, the APACHE II score was 22.05±6.32, and the SOFA score was 8.26±2.48. While 66.7% (n:80) of the patients included in the study died, the length of stay in the ICU was 12.85±10.23 days. The parameters and demographic characteristics of the patients before and after dialysis are shown in Table 1.

**Table 1:** The demographic, clinical, and laboratory characteristics of the groups during admission to the intensive care unit IHD: Intermittent Hemodialysis, CRRT: Continuous Renal Replacement Therapy, SAPS: Simplified Acute Physiology Score, APACHE: Acute Physiology and Chronic Health Evaluation, SOFA: Sepsis-related Organ Failure Assessment, ICU: Intensive Care Unit

Parameters	All Patients (n=120)	IHD (n=80)	CRRT (n=40)	p
Sex: Female; Male	74 (61.7%); 46 (38.3%)	49 (61.3%); 31 (38.7%)	25 (62.5%) 15 (37.5%)	0.894
Age	62.90±13.64	61.41±15.24	65.90±9.13	0.089
SAPS-II	45.05±12.76	43.35±11.11	48.47±15.13	0.038
APACHE II Scores	22.05±6.32	21.06±5.51	24.02±7.38	0.015
SOFA Scores	8.26±2.48	7.91±2.49	8.97±2.35	0.027
Pre-Dialysis				
Mean Arterial Pressure	79.025±15.29	81.75±16.23	73.65±11.62	0.006
Urea (mg/dL)	159.76±66.15	167.18±70.17	144.92±55.13	0.082
Creatinine (mg/dL)	4.09±1.94	4.44±2.09	3.39±1.40	0.005
Sodium (mmol/L)	134.97±6.49	135.08±6.13	134.75±7.24	0.790
Potassium (mmol/L)	4.93±0.94	5.02±1.07	4.73±0.52	0.110
Calcium (mg/dL)	8.36±1.35	8.25±1.42	8.59±1.19	0.198
pH	7.31±0.09	7.29±0.10	7.35±0.07	<0.001
Lactate (mmol/L)	2.33±1.97	2.23±2.22	2.52±1.37	0.448
Bicarbonate (mmol/L)	20.00±4.38	19.59±4.55	20.81±3.95	0.151
Post-Dialysis				
Mean Arterial Pressure	74.49±13.67	75.17±14.67	73.13±11.45	0.442
Urea (mg/dL)	121.08±50.63	115.70±51.21	131.85±48.29	0.100
Creatinine (mg/dL)	3.24±1.46	3.23±1.51	3.27±1.39	0.868
Sodium (mmol/L)	135.80±5.68	136.35±4.73	134.72±7.16	0.140
Potassium (mmol/L)	4.48±0.89	4.48±1.02	4.48±1.02	0.994
Calcium (mg/dL)	8.37±1.01	8.35±1.05	8.43±0,94	0.696
pH	7.32±0.13	7.33±0,14	7.30±0,11	0.338
Lactate (mmol/L)	3.04±3.04	2.88±1.41	3.37±2.10	0.403
Bicarbonate (mmol/L)	21.73±4.14	21.72±4.53	21.75±3.28	0.975
Non-survivor Survivor	80 (66.7%); 40 (33.3%)	53 (66.3%); 27 (33.7%)	27 (67.5%); 13 (32.5%)	0,891
Length of Stay in ICU (days)	12.85±10.23	14.02±10.31	10.50±9.77	0.075

The IHD group and the CRRT group were compared, and no difference was found between the two groups in terms of gender (p:0.894). The mean age in the IHD group was 61.41±15.24 years while the mean age in the CRRT group was 65.90±9.13, and there was no statistical difference between the groups (p:0.089). The SAPS-II, APACHE II, and SOFA scores were significantly higher in the CRRT group than in the IHD group (p:0.038, p: 0.015, p:0.027, respectively). When the pre-dialysis parameters of the groups were compared, MAP and creatinine levels were found to be statistically significantly lower and pH values ​​higher in the CRRT group (p: 0.006, p:0.005, p:<0.001, respectively). In addition, although urea, sodium, and potassium levels were lower and lactate and bicarbonate levels were higher in the CRRT group before dialysis, no difference was found between the groups. There was no significant difference between the groups in all parameters measured after dialysis (p>0.05). Although the length of stay in the ICU was shorter in the CRRT group, it was not statistically significant (p:0.075). When mortality was evaluated, 66.3% of the IHD group and 67.5% of the CRRT group died (p:0.891).

**Figure 1:**
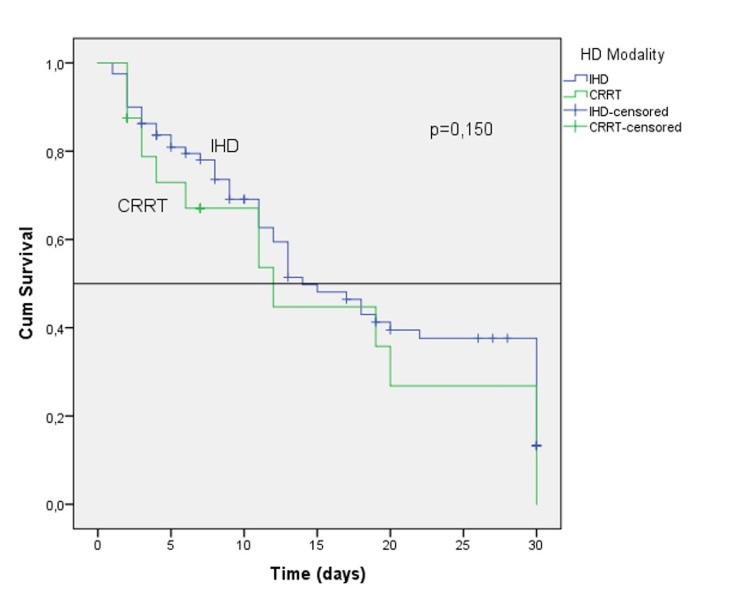
Kaplan-Meier survival analysis for hemodialysis modality

The patients who underwent RRT were evaluated by Kaplan-Meier survival analysis; no difference was found between the groups (p:0.150) (Figure 1). According to Cox regression analysis, the SAPS-II, APACHE II, and SOFA scores affected mortality in both univariate and multivariate analyses while age, gender, and RRT modalities were not associated with mortality (Table 2).

**Table 2:** Univariate and multivariate Cox Regression analysis results to evaluate 30-day mortality risk SAPS: Simplified Acute Physiology Score, APACHE: Acute Physiology and Chronic Health Evaluation, SOFA: Sepsis-related Organ Failure Assessment, RRT: Renal Replacement Therapy, CRRT: Continuous Renal Replacement Therapy

	Univariate			Multivariate		
Parameters	OR	95%CI	p	OR	95% CI	p
Age	1.01	0.99 - 1.03	0.183	1.01	0.99 - 1.03	0.116
Sex (Male)	1.19	0.75 - 1.89	0.458	1.32	0.77 - 2.26	0.314
SAPS-II	1.04	1.02 -1.06	<0.001	1.03	1.01 - 1.06	0.007
APACHE II Scores	1.08	1.04 - 1.11	<0.001	1.05	1.01 - 1.09	0.028
SOFA Scores	1.19	1.10 - 1.30	<0.001	1.13	1.02 - 1.26	0.019
RRT Modality (CRRT)	1.36	0.85 - 2.16	0.198	0.87	0.51 - 1.50	0.616

Changes in parameters before and after dialysis were evaluated (Table 3). There was a greater decrease in MAP after dialysis in the IHD group (p<0.001). While there was a decrease in urea, creatinine, and potassium clearance in both groups after dialysis, this decrease was statistically more significant in the IHD group compared to the CRRT group (p<0.001, p<0.001, p:0.005, respectively).

**Table 3:** Changes in values after dialysis and before dialysis and statistical analysis IHD: Intermittent Hemodialysis, CRRT: Continuous Renal Replacement Therapy

Parameters	IHD (n=80)	CRRT (n=40)	p
Mean Arterial Pressure	-6.57±0.46	-0.52±1.57	<0.001
Urea (mg/dL)	-51.48±4.19	-13.075±4.99	<0.001
Creatinine (mg/dL)	-1.21±1.27	-0.11±0.08	<0.001
Sodium (mmol/L)	1.26±0.49	-0.02±0.40	0.03
Potassium (mmol/L)	-0.54±0.91	-0.25±0.06	0.005
Calcium (mg/dL)	0.09±0.12	-0.16±0.68	0.044
pH	0.04±0.12	-0.04±0.15	<0.001
Lactate (mmol/L)	0.64±0.38	0.85±0.30	0.068
Bicarbonate (mmol/L)	2.13±0.45	0.93±0.29	0.025

When the blood gas was evaluated after dialysis, there was no increase in pH value in the CRRT group while a statistically significant increase was found in the pH value in the IHD group (<0.001). While bicarbonate levels increased in both groups, this increase was more significant in the IHD group (p:0.025).

## Discussion

AKI is part of the multiple organ dysfunction syndrome in critically ill patients and is a common condition in ICU patients. RRT is the cornerstone of the treatment of critically ill AKI. Despite RRT, it has a high mortality rate. Many studies have examined the modalities of RRT applied in critically ill patients with AKI [6-7,9]. In these studies, some parameters were compared between IHD and CRRT.

Scoring systems, such as APACHE II, SOFA, and SAPS-II, which have critical importance in the follow-up of patients and predict mortality, are used in ICUs. In the literature, these scoring systems were evaluated in critically ill patients who developed AKI and required RRT. In the CONVINT study, no difference was found between the IHD (n:128) group and the CRRT (n:122) group in terms of APACHE II, SOFA, and SAPS-II scores (p:0.79, p:0.66, p:0.34, respectively) [9]. On the other hand, in the OUTCOMEREA study that included 1360 patients (CRRT: 544 patients, IHD: 816), the SOFA score in the CRRT group (p<0.001) [3], and the APACHE II score in the CRRT group (p<0.001) in the observational study by Rauf et al. was found to be significantly higher [10]. In another study, Bonnassieux et al. evaluated 58,605 patients and found a higher SAPS-II score (p<0.001) in the CRRT group [11]. In our study, unlike other studies, we evaluated all three scoring systems between groups and found APACHE II, SOFA, and SAPS-II to be significantly higher in the CRRT group (p:0.015, p:0.027, p:0.038, respectively).

Most studies and meta-analyses have compared CRRT with IHD in terms of mortality and length of hospital stay in ICU patients who develop AKI and undergo RRT. In the meta-analysis of 21 studies by Nash et al., the superiority of the groups to each other was not shown in terms of 30-day mortality and ICU stay [12]. Again, in the CONVINT study, 14-day and 30-day mortality were evaluated, and similar to other studies, no difference was found between the groups. In terms of length of stay in the ICU, although the length of hospital stay was lower in the CRRT group, no statistical difference was found [9]. In another study, there was no significant difference in mortality in the CRRT and IHD groups at the end of 90 days [6]. We evaluated the 30-day mortality in our study and found no difference between these two RRT modalities. When we look at the length of stay in the ICU, similar to the CONVINT study, although the length of stay in the CRRT group was lower, it was not significant (p:0.075). We also evaluated the factors affecting 30-day mortality in our study. We observed that age, gender, and RRT modality did not affect mortality while high SAPS-II, APACHE II, and SOFA scores increased the risk of mortality.

Both CRRT and IHD similarly provide adequate metabolic control. However, the speed of reaching it is different. In most ICUs, the choice of RRT modality is usually determined by the patient's hemodynamic status. The KDIGO guideline recommends that CRRT should be preferred primarily for hemodynamically unstable patients [8]. In our study, we generally preferred CRRT in hemodynamically unstable patients. When we evaluated MAP before dialysis, we found that it was significantly lower in the CRRT group than in the IHD (p: 0.006). When the groups were compared in terms of clinical outcomes in the studies performed, no significant differences were shown between those who underwent IHD and CRRT [6-7,13]. However, it is recommended to prefer IHD for rapid correction of electrolyte disorders such as hyperkalemia [4,6,14]. In our study, when we examined the variability in laboratory parameters between the groups before and after RRT, although we observed improvements in urea, creatinine, sodium, potassium, calcium, bicarbonate, and pH values ​​in both groups, this improvement was statistically significantly higher in the IHD group. When we evaluated the variability in MAP, we found that MAP before and after dialysis decreased more in the IHD group than in the CRRT group. We attributed this to the fact that CRRT provides better hemodynamic stability by providing slow but continuous removal of fluid and solute load.

The limitation of this study is that the study was single-centered. Multicenter studies may provide stronger results.

## Conclusions

In conclusion, in our study, we could not find a statistically significant difference between CRRT and IHD in terms of mortality and ICU stay in critically ill patients who underwent RRT for AKI in the ICU. Although adequate metabolic control was achieved with both RRT modalities, this improvement was more significant in the IHD group in our study. The reduction in MAP was greater in the IHD group. Both modalities have advantages and disadvantages in this patient population. Since there is no superiority to each other in terms of mortality and length of stay in the ICU, which modality should be used should be evaluated on a patient basis.
